# Change in heart rate variability precedes the occurrence of periodic leg movements during sleep: an observational study

**DOI:** 10.1186/1471-2377-13-139

**Published:** 2013-10-06

**Authors:** Taeko Sasai, Masato Matsuura, Yuichi Inoue

**Affiliations:** 1 Department of Somnology, Tokyo Medical University, 1-24-6, Yoyogi, Shibuya-ku Tokyo, Japan; 2 Japan Somnology Center, Neuropsychiatric Research Institute, Tokyo, Japan; 3 Department of Life Sciences and Bio-informatics, Division of Biomedical Laboratory Sciences, Graduate School of Health Sciences, Tokyo Medical and Dental University, Tokyo, Japan

## Abstract

**Background:**

Several reports have described that individual periodic leg movements during sleep (PLMS) activities are associated with autonomic nervous system activity occurring shortly before each PLMS activity. Nevertheless, no study has investigated dynamic changes of autonomic nervous system activity before the onset of PLMS. This study detected changes in heart rate variability (HRV) at the onset of the period with PLMS using complex demodulation method.

**Methods:**

This study enrolled 14 patients diagnosed as having idiopathic PLMS disorder (PLMD). In periods with and without PLMS during sleep stage 2, HRV-related variables and the spectral power of fluctuation of a high frequency (HF) band (FHFB) were analyzed and compared. The changes of those parameters during transition from the period without PLMS to that with PLMS were explored.

**Results:**

Spectral power in the low frequency (LF) band and very low frequency (VLF) band were higher in the period with PLMS. Additionally, the average frequency in FHFB was higher. The frequency in this band fluctuated during the period with PLMS with remarkable elevation of FHFB. Moreover, spectral powers in FHFB, LF, and VLF were remarkably elevated shortly before the beginning of the period with PLMS (FHFB, -65 s; LF, -53 s; and VLF, -45 s).

**Conclusions:**

Elevation of sympathetic nervous system activity and mean frequency fluctuation in an HF band can occur several tens of seconds before the period with PLMS. Dynamic changes in the autonomic nervous system activity might be related to the vulnerability to PLMS occurrence during the night.

## Background

Periodic leg movements during sleep (PLMS) are often associated with autonomic arousal, cortical arousal, and awakening [1-4]. PLMS comorbid with RLS has also been inferred to play a role in the formation of nocturnal hypertension [5] and increased cardiovascular risk [6].

Although PLMS are likely to occur during the sleep stage 2 [7-9], patients with PLMS generally have periods with frequent PLMS and also PLMS-free periods, even during the same sleep stage in the course of a single night [10]. Nevertheless, the trigger of the initiation of the period with frequent PLMS has not been elucidated yet. During the last decade, reports have described that changes in heart rate or cortical activity occur a few seconds before the onset of each instance of PLMS activity [1,4,11-14]. Moreover, recent reports have described the relation between PLMS and heart rate variability (HRV) [15,16]. These previous studies indicated that sympathetic nervous activity increased shortly before individual PLMS activity or during the period with PLMS. Nevertheless, no report in the relevant literature describes an investigation of dynamic change in heart rate variability through the period preceding the occurrence of PLMS.

This study was conducted to explore the changes in HRV in transition from the period in which PLMS are absent to the period with frequent PLMS using time-frequency domain analysis, and complex demodulation method, which has high temporal resolution to delineate time-dependent fluctuations in both the amplitude and frequency of non-stationary and long-period data [17,18].

## Methods

### Subjects

The Ethics Committee of the Neuropsychiatric Research Institute approved this retrospective study. Informed consent was obtained from all participants. Among patients who visited the Yoyogi Sleep Disorder Clinic and underwent nocturnal polysomnography (n-PSG) during October 2010 – March 2011, 14 patients with PLMD were enrolled in this study (mean age, 45.9 ± 11.5 yr; M:F = 9:5). The inclusion criteria applied to patients were the following: 1) diagnosed as idiopathic PLMD according to the International Classification of Sleep Disorders Second Edition [19]; 2) apnea–hypopnea index < 5/hr; 3) absence of any pathological arrhythmia; 4) absence of any muscular or neurologic disease; and 5) not taking medication that might affect autonomic nervous system activity (Table 1).

**Table 1:** Patient characteristics and sleep variables of subject patients with PLMD

	
Gender (M:F)	9:5
Age (yr)	45.9 ± 11.5
Epworth Sleepiness Scale (points)	12.8 ± 4.1
Apnea–hypopnea index (n/hr)	1.7 ± 0.1
Wake after sleep onset (%SPT)	6.1 ± 4.4
Stage 1 (%SPT)	11.7 ± 5.0
Stage 2 (%SPT)	56.9 ± 7.5
Stage 3 + 4 (%SPT)	4.4 ± 5.5
Stage REM (%SPT)	21.0 ± 4.1
Arousal index (n/hr)	13.9 ± 5.3
PLMS index (n/hr)	34.1 ± 14.8
PLMS related arousal index (n/hr)	4.0 ± 2.3
Sleep latency (min)	7.2 ± 8.0
Sleep efficiency (%)	91.7 ± 3.7

Values are expressed as mean ± SD, except for gender distribution.

PLMD, periodic leg movement disorder; SPT, sleep period time; REM, rapid eye movement; PLMS, periodic leg movement during sleep.

### Nocturnal polysomnography and scoring

Using a standard system (Alice 5; Respironics Inc., Murrysville, PA, USA) with video monitoring of patient behavior, we performed diagnostic n-PSG recordings and measurements including four channels of the scalp EEG (C3/A2, C4/A1, O1/A2, O2/A1), two electrooculograms (EOG), submental electromyogram (EMG), electrocardiogram, nasal/oral airflow, oximetry sensor for percutaneous oxygen saturation (SpO_2_) recording, a microphone for snoring sounds, chest/abdominal respiratory effort assessment, and anterior tibialis electromyogram for leg movements (bipolar derivations with two electrodes placed 3 cm apart on the belly of the anterior tibialis muscle of right and left legs). Sleep stages were scored according to the criteria set by Rechtschaffen and Kales [20]. EEG arousal was scored according to the criteria set by the American Sleep Disorders Association [21]. Episodes of PLMS were defined as leg movements with an amplitude increase of 8 μV above the baseline value, a duration of 0.5–10 s, an interval between two consecutive movements of 5–90 s, and a minimum of four consecutive movements [22,23].

### Data analysis

In patients with PLMS, HRV was analyzed in stable stage 2 NREM sleep during 10-min periods in which PLMS appeared consecutively and frequently (with PLMS; 104 periods in total; mean ± standard deviation (SD) for the number of analyzed periods, 7.4 ± 2.3; range of duration of analyzed periods, 10–12.5 min) and during 10-min periods in which PLMS was completely absent (without PLMS; 86 periods in total; mean ± SD for number of analyzed periods, 6.1 ± 1.5; range of duration of analyzed periods, 10–13.5 min). HRV was also analyzed during 15-min periods in transition from a period without PLMS to one with PLMS (from 10 min before to 5 min after the beginning of PLMS) during the stable sleep stage 2 (14 periods in all). For these eligible periods, stable sleep refers to periods with neither a change of the sleep stage from stage 2 nor any EEG arousal. Periods eligible for analyses were those which were free of movement artifacts on the EEG signal, respiratory events, and cortical EEG arousals. Such periods were selected carefully by a board-certified sleep technician.

**Figure 1:**
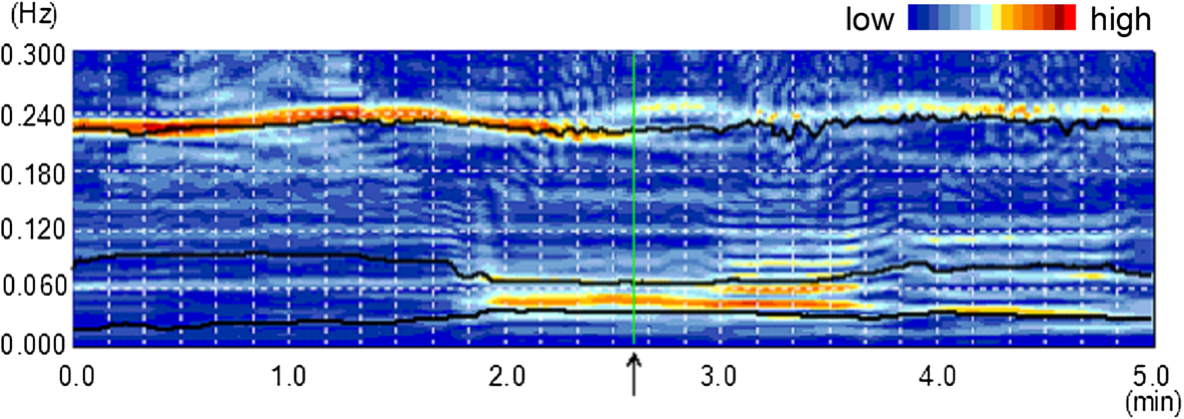
**Example of DSA representation in transition from the period without PLMS to that with PLMS.** An arrow on the time axis indicates the beginning of the period with PLMS. Solid lines represent the mean frequency of each frequency bands (HF, LF, VLF); DSA, density spectrum array; PLMS, periodic leg movements during sleep; HF, high frequency; LF, low frequency; VLF, very low frequency.

Measures of HRV were performed in both time and frequency domains. For each period, time-dependent changes in the amplitude of LF, HF, and VLF were assessed using complex demodulation method (CDM) [17,18], which can continuously assess frequency shifts and time-dependent changes in the amplitude of the rhythmic components existing in predefined frequency bands. In fact, CDM is a nonlinear time domain method of time series analysis that is particularly suitable for the investigation of nonstationary and unstable oscillations. The CDM process comprises four steps. First, the spectral region of interest is shifted to zero frequency by forming a product, throughout the record, of the original signal and a complex sinusoid at a reference frequency (the center frequency of the spectral region of interest). Second, the resultant complex signal is low-pass filtered to exclude frequency components other than those around zero. Third, the real and imaginary parts of the low-pass filtered signal are converted to a polar form, yielding the amplitude and phase, as a function of time, of the component identified at or near the reference frequency. Finally, because the slope of the phase vs. time curve represents deviation of instantaneous operative frequency from the reference frequency, the time series of the actual frequency within the spectral region of interest is calculated from the phase [17,18]. Data were analyzed using a computer program with 200 ms time resolution capability (R-R Interval CDM Pro Analysis; Norupro Light Systems Inc., Tokyo, Japan). In this program, a density spectrum array (DSA) was created for 3D demonstration of amplitude for each frequency band with high-temporal resolution (1 s) (Figure 1). The spectral power in each frequency band of VLF (< 0.04 Hz), LF (0.04–0.15 Hz), and HF (0.15–0.4 Hz) was obtained by calculating the average amplitude (ms) for all eligible periods. When we checked DSA data visually, fluctuation of the mean frequency was recognized only in the HF band, although the mean frequency of the HF band in a healthy person is mostly stable during NREM sleep [24]. Therefore, for evaluating the fluctuation of the HF band frequency, the frequency of the fluctuation for each time point (1 s) obtained using the program described above was converted to a value eligible for CDM analysis using a program (HRV LOG-Pro-DSA Analysis; Norupro Light Systems Inc., Tokyo, Japan).

To detect changes in EEG activity, which are reportedly associated with changes in HRV-related variables or occurrence of PLMS [25-28], we conducted EEG spectral analysis in transition from periods without PLMS to those with PLMS. After adjusting the start time of the period with PLMS, the mean EEG power was calculated in each point to detect changes in EEG spectral power at the beginning of the period with PLMS. This quantitative EEG evaluation was performed using fast Fourier transform (FFT) on the periods. The FFT on 4-s epochs with a Hamming window yielding 0.25 Hz of spectral resolution was performed on C3/A2 and C4/A1 derivations using a computer program (CSA play analysis; Norupro Light Systems, Tokyo, Japan).

### Statistical analysis

Comparison of HRV-related variables from periods with and without PLMS was conducted using Wilcoxon signed-rank tests for non-parametric comparison using software (Statistical Package for the Social Sciences, SPSS, ver. 17.0, 2009; IBM, Tokyo, Japan). The significance level was set as *p* < 0.05. Points of increase in the HRV-related variables were determined using confidence intervals for respective parameters. The interval included values within three standard errors from the mean baseline mean value, corresponding to a nominal significance of *p* < 0.05. Differences from baseline were considered significant when time periods in which a parameter exceeded the confidence interval were longer than 1 s. A point of increase was determined as the starting time of the time period. A repeated-measures ANOVA was conducted to clarify the presence of PLMS-related changes in EEG powers in each frequency band.

## Results

### HRV-related variables in the period with PLMS

Table 2 presents a comparison of the HRV-related variables obtained in periods with and without PLMS. Significant differences were found in VLF power (*Z* = −3.296, *p* < 0.01, *r* = 0.88), LF power (*Z* = −3.296, *p* < 0.01, *r* = 0.88), LF/HF (*Z* = −3.296, *p* < 0.01, *r* = 0.88), and mean frequency of FHFB (Z = −2.856, *p* < 0.01, *r* = 0.76). During the period with PLMS, VLF power, LF power, LF/HF, and mean frequency of FHFB were significantly higher than they were in the period without PLMS.

**Table 2:** Heart rate variation during periods with and without PLMS

	**Periods with PLMS**	**Periods without PLMS**	***p***	***r***
VLF power (ms)	23.1 ± 8.5	13.3 ± 4.3	< 0.01	0.88
LF power (ms)	16.4 ± 6.6	7.7 ± 2.8	< 0.01	0.88
HF power (ms)	10.2 ± 6.5	11.8 ± 8.8	n.s.	-
LF/HF	2.25	1.02	< 0.01	0.88
mean frequency in VLF (Hz)	0.030 ± 0.001	0.028 ± 0.001	n.s.	-
mean frequency in LF (Hz)	0.075 ± 0.004	0.075 ± 0.004	n.s.	-
mean frequency in HF (Hz)	0.220 ± 0.016	0.223 ± 0.020	n.s.	-
mean frequency in FHFB (Hz)	0.045 ± 0.090	0.038 ± 0.007	< 0.01	0.76
mean frequency of PLMS (Hz)	0.035 ± 0.012	-	-	-

Values are expressed as mean±SD, except for LF/HF.

PLMS, periodic leg movement during sleep; VLF, very low frequency; LF, low frequency: HF, high frequency; FHFB, fluctuation of a HF band; n.s., not significant.

### Changes in HRV-related variables and quantitative EEG variables in transition from periods without PLMS to those with PLMS

**Figure 2:**
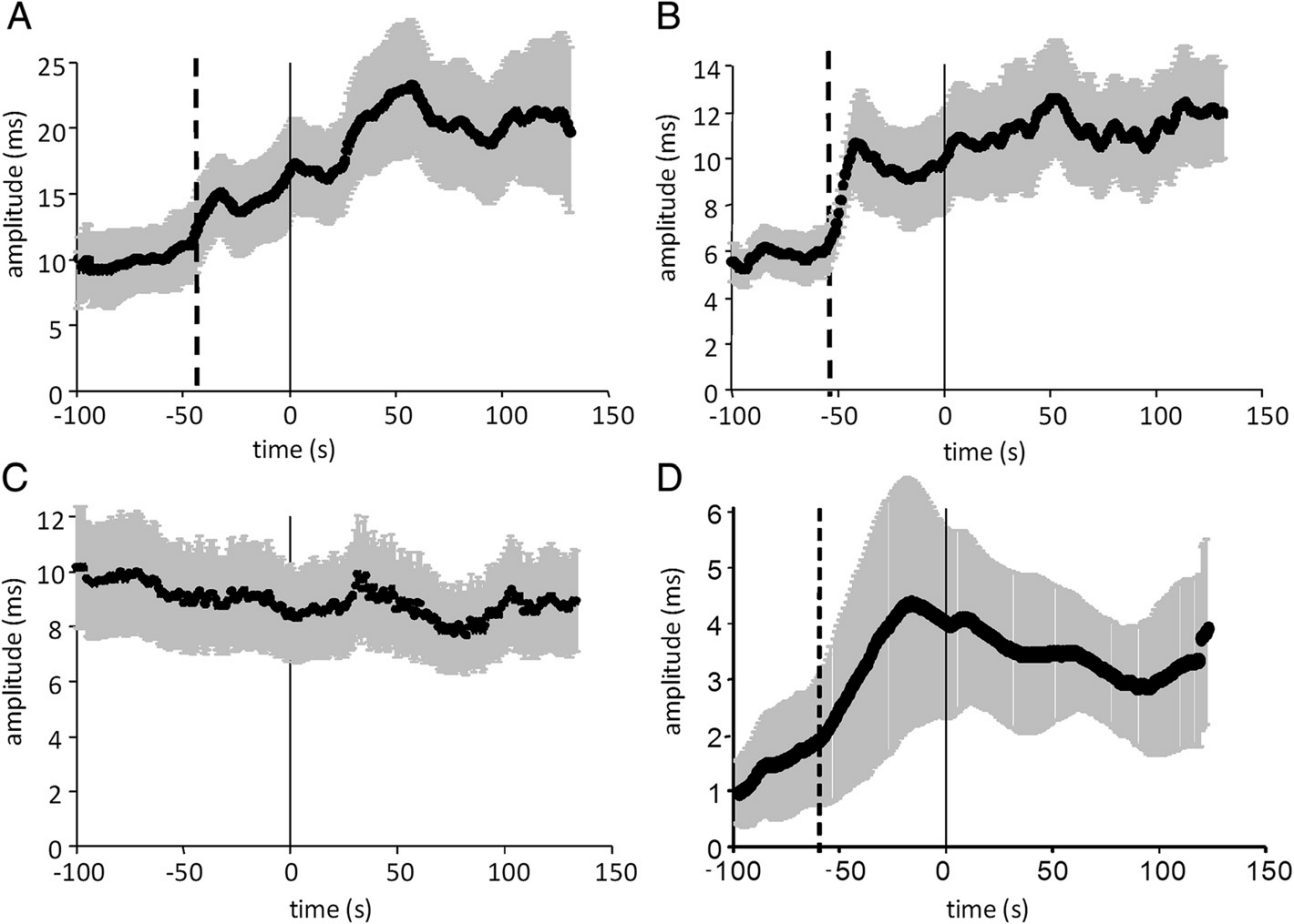
**Changes in the HRV-related variables in transition from periods without PLMS to those with PLMS. (A)** VLF, very low frequency; **(B)** LF, low frequency; **(C)** HF, high frequency; **(D)** FHFB, fluctuation of a HF band; PLMS, periodic leg movements during sleep. Data are expressed as means ± standard errors. Note: “0” on the time axis signifies the beginning of the period with PLMS.

**Figure 3:**
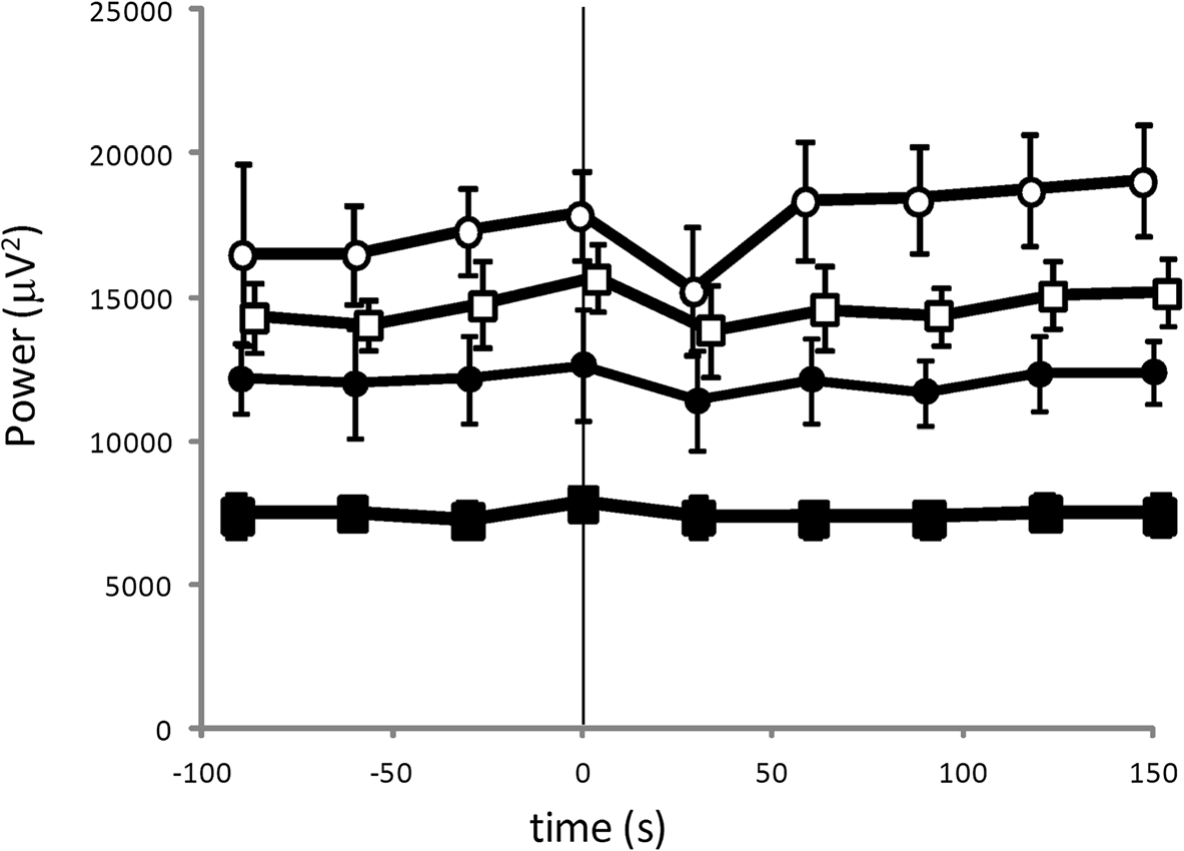
**Changes in the EEG spectral powers in transition from periods without PLMS to those with PLMS: filled circles, alpha frequency band; filled squares, beta frequency band; open circles, delta frequency band; open squares, theta frequency band; PLMS, periodic leg movements during sleep.** Data are expressed as mean ± standard error. Note: “0” on the time axis signifies the beginning of the period with PLMS.

Figure 2A–2D show changes in VLF power, LF power, HF power, and FHFB power in transition from the period without PLMS to the period with PLMS. VLF power increased 45 s before the beginning of the period with PLMS (Figure 2A). LF power also increased 53 s before the beginning of the period with PLMS (Figure 2B). In contrast, HF power showed no remarkable change in the transitional zone from the period without PLMS to the period with PLMS (Figure 2C). However, the FHFB power increased remarkably 65 s before the beginning of the period with PLMS (Figure 2D). No significant change in EEG spectral power synchronized with the beginning of the period with PLMS was observed in any frequency band in the central region (Figure 3).

## Discussion

This report is the first to describe a study using CDM to explore dynamic changes in HRV preceding the beginning of a period with PLMS. CDM, which has high temporal resolution and high frequency resolution for detecting dynamic changes in HRV, even in a non-stationary and long period, provides data revealing time-dependent changes in amplitude of several undefined frequency components on a continual basis [17,18]. Several earlier reports have described that the heart rate increases a few seconds before individual leg movements occur [4,11-13]. Sforza et al., from results of a study using fast Fourier transformation (FFT) and wavelet transform, reported significant increases in the VLF power, LF power, and LF/HF in the period with PLMS compared to the period without PLMS [15]. Walters et al. also reported higher VLF power and LF/HF rate in the period with PLMS [29]. Consistent with results of those earlier studies, VLF power, LF power, and LF/HF power showed a remarkable increase in the period with PLMS compared to the period without PLMS in this study. In addition, the frequency of PLMS was in the VLF band. These findings indicate that a shift in sympathovagal balance to sympathetic dominance in the period with PLMS is influenced strongly by an increase in sympathetic activation in response to PLMS [15]. Moreover, the VLF component might be associated with the rhythm formation process of PLMS. However, temporal information is unobtainable using FFT because it only provides frequency and amplitude data that are averaged over the entire length of the time series, based on the assumption that the system is stationary during the analyzed period. In addition, wavelet transformation, which has low temporal resolution, is unable to detect waves with small amplitude. Consequently, these techniques used in previous studies might be inappropriate for assessing sudden, time-dependent changes in the amplitudes of particular frequency bands. Therefore, changes in autonomic function, which might be related to the vulnerability to the occurrence of PLMS occurring in the transition zone to the period with PLMS, have not been explored to date.

The most striking result of this study was that the VLF power and LF power increased remarkably several tens of seconds before the beginning of the period with PLMS. These results suggest that a shift in the sympathovagal balance to sympathetic dominance begins long before the start of the period with PLMS. In contrast, HF power did not change before the beginning of the period with PLMS. However, the frequency of the HF band started to fluctuate concurrently with the respective increases of VLF power and LF power. Reportedly, the mean frequency of the HF band and its oscillation are lower during sleep than during wakefulness, and sympathovagal balance shifts to parasympathetic dominance during sleep [24]. Considering these facts, the fluctuation of a HF band during sleep stage 2 in our subject patients with PLMD might indicate that parasympathetic nervous activity becomes unstable before the period with PLMS. Several previous reports have demonstrated that cyclic alternating patterns (CAP) or changes in the EEG spectrum strongly influence changes in HRV-related variables in association with PLMS [25-28]. In the present study, however, EEG spectral powers in the central region did not change at the beginning of the period with PLMS. Therefore, the observed dynamic changes in HRV-related variables were not thought to be influenced by changes in EEG spectral power. A recent report has described that the presence of RLS or PLMS presents a risk of cardiovascular disease. However, cardiovascular diseases such as congestive heart failure and stroke are frequently associated with PLMS possibly because of vascular changes in the central nervous system and periphery [6,30-33]. In addition, some previous reports have described that sympathetic hyperactivity might exacerbate RLS/PLMS symptoms [34,35]. Considering these facts, the sympathovagal balance towards sympathetic dominance occurring several tens of seconds before the start of the appearance of PLMS, which might be related with vulnerability to the occurrence of PLMS, might be associated with the future development of cardiovascular diseases.

This paper presents some limitations. First, it was necessary to exclude many data because of movement artifacts on the EEG signal, respiratory events, and cortical arousal. For that reason, patients with sufficient data were few. A study conducted with increased sample size might reinforce the present results in a future study. For example, such a future study should address the impact of severity of PLMS on HRV change. A second important limitation is the lack of data from healthy controls. A previous study revealed that experimental sleep fragmentation is not associated with an increase in the frequency of PLMS in normal young adults [36]. Comparison of the patterns of changes in HR variables between subjects with PLMS and healthy controls using CDM must be undertaken in further studies. Third is a lack of data from periods with a shift towards sympathetic dominance that is not followed by PLMS. Future studies should analyze data of this period to clarify whether shifts to sympathetic dominance can trigger PLMS occurrence. Fourth, unfortunately, CAP phases and K-complexes could not be excluded from the analyzed periods because exclusion of periods with these transient EEG events engenders a lack of length of periods for the present analysis. These transient EEG events are regarded as synchronous with the occurrence of each PLMS event [27]. Future studies will examine changes in HRV-related variables before the beginning of period with PLMS. These changes should be explored while controlling for these transient EEG events.

## Conclusions

Not only sympathetic hyperactivity, but also fluctuation of parasympathetic nervous activity occurred several tens of seconds before the beginning of the period with PLMS. These dynamic changes in the autonomic nervous system activity that occur before the beginning of the period with PLMS are inferred to be related with the mechanism of PLMS occurrence.

## Abbreviations

AASM: American Academy of Sleep Medicine; AHI: Apnea hypopnea index; CDM: Complex demodulation method; HRV: Heart rate variability; LF: Low frequency; HF: High frequency; FHFB: Fluctuation of a high frequency band; DSA: Density spectrum array; VLF: Very low frequency; RLS: Restless legs syndrome; EEG: Electroencephalogram; EMG: Electromyogram; EOG: Electrooculogram; ICSD-2: International classification of sleep disorders, second revision; NREM: Non-rapid eye movement; PLMS: Periodic leg movement during sleep; PSG: Polysomnography; REM: Rapid eye movement; SpO2: Percutaneous oxygen saturation.

## Competing interests

The authors declare that they have no competing interests.

## Authors’ contributions

TS, a principal investigator and guarantor who designed and conceptualized the study, analyzed and interpreted the data, and drafted and revised the manuscript for intellectual content. MM revised the manuscript. YI designed and conceptualized the study and revised the manuscript for intellectual content. All authors read and approved the final manuscript.

## Pre-publication history

The pre-publication history for this paper can be accessed here:

http://www.biomedcentral.com/1471-2377/13/139/prepub
